# Modeling the limits of detection for antimicrobial resistance genes in agri-food samples: a comparative analysis of bioinformatics tools

**DOI:** 10.1186/s12866-023-03148-6

**Published:** 2024-01-20

**Authors:** Ashley L. Cooper, Andrew Low, Alex Wong, Sandeep Tamber, Burton W. Blais, Catherine D. Carrillo

**Affiliations:** 1 Research and Development, Ottawa Laboratory (Carling), Canadian Food Inspection Agency, Ottawa, ON Canada; 2 Department of Biology, Carleton University, Ottawa, ON Canada; 3 Microbiology Research Division, Bureau of Microbial Hazards, Health Canada, Ottawa, ON Canada

**Keywords:** Metagenomics, Antimicrobial resistance, Sequence coverage, Limit of detection

## Abstract

**Background:**

Although the spread of antimicrobial resistance (AMR) through food and its production poses a significant concern, there is limited research on the prevalence of AMR bacteria in various agri-food products. Sequencing technologies are increasingly being used to track the spread of AMR genes (ARGs) in bacteria, and metagenomics has the potential to bypass some of the limitations of single isolate characterization by allowing simultaneous analysis of the agri-food product microbiome and associated resistome. However, metagenomics may still be hindered by methodological biases, presence of eukaryotic DNA, and difficulties in detecting low abundance targets within an attainable sequence coverage. The goal of this study was to assess whether limits of detection of ARGs in agri-food metagenomes were influenced by sample type and bioinformatic approaches.

**Results:**

We simulated metagenomes containing different proportions of AMR pathogens and analysed them for taxonomic composition and ARGs using several common bioinformatic tools. Kraken2/Bracken estimates of species abundance were closest to expected values. However, analysis by both Kraken2/Bracken indicated presence of organisms not included in the synthetic metagenomes. Metaphlan3/Metaphlan4 analysis of community composition was more specific but with lower sensitivity than the Kraken2/Bracken analysis. Accurate detection of ARGs dropped drastically below 5X isolate genome coverage. However, it was sometimes possible to detect ARGs and closely related alleles at lower coverage levels if using a lower ARG-target coverage cutoff (< 80%). While KMA and CARD-RGI only predicted presence of expected ARG-targets or closely related gene-alleles, SRST2 (which allows read to map to multiple targets) falsely reported presence of distantly related ARGs at all isolate genome coverage levels. The presence of background microbiota in metagenomes influenced the accuracy of ARG detection by KMA, resulting in *mcr*-1 detection at 0.1X isolate coverage in the lettuce but not in the beef metagenome.

**Conclusions:**

This study demonstrates accurate detection of ARGs in synthetic metagenomes using various bioinformatic methods, provided that reads from the ARG-encoding organism exceed approximately 5X isolate coverage (i.e. 0.4% of a 40 million read metagenome). While lowering thresholds for target gene detection improved sensitivity, this led to the identification of alternative ARG-alleles, potentially confounding the identification of critical ARGs in the resistome. Further advancements in sequencing technologies providing increased coverage depth or extended read lengths may improve ARG detection in agri-food metagenomic samples, enabling use of this approach for tracking clinically important ARGs in agri-food samples.

**Supplementary Information:**

The online version contains supplementary material available at 10.1186/s12866-023-03148-6.

## Background

Antimicrobial use in medicine and agriculture is a potential driver of antimicrobial resistance (AMR) dissemination [1]. Many environments including plants, animals, food, and water sources can function as routes for transfer of AMR genes (ARGs) within and between bacterial populations [2, 3]. Food production connects many of these habitats, potentially furthering the spread of both AMR and pathogenic bacteria [3].

Food production occurs along a continuum from agricultural and manufacturing production processes to distribution and consumption, with multiple points for the entry of microbial contaminants [4]. Food-testing practices for detecting bacterial pathogens traditionally require sampling of food products and production facilities followed by enrichment and culturing for organisms of interest. However, these methods are time consuming, labor intensive, and only target and identify specific pathogenic bacteria (e.g. *Salmonella* and *Listeria monocytogenes*), which may not be the principal reservoirs for clinically important ARGs. In contrast, other genera commonly found in agri-food samples, such as *Citrobacter*, *Enterobacter*, *Hafnia*, *Klebsiella*, and *Proteus* more often exhibit AMR of concern [5–7].

AMR detection is achievable using a variety of different phenotypic and molecular methods [8]. Similar to pathogen detection, culture-based approaches are often laborious, species-specific, and exclude unculturable isolates [9–11]. Molecular methods that target known ARGs are generally quicker and more cost-effective. Common techniques include PCR, quantitative or real-time PCR (qPCR), hybridization techniques, high resolution melting curve analysis, and matrix-assisted laser desorption ionization-time of flight mass spectrometry (MALDI-TOF MS) [12–17]. Yet, these approaches are also limited to analysis of well-studied organisms or ARGs and are not always useful for screening a large number of targets. Additional limitations arise due to the large number of ARG allelic variants, making development of all-encompassing assays for a single gene target almost impossible. In addition, discovery of novel ARGs may result in the need to design additional assays and re-analyse samples.

Metagenomic sequencing has the potential to bypass the limitations of culture-based and other molecular techniques, while also enabling evaluation of a sample’s microbial diversity [18]. Yet, this approach is not without its own intrinsic limitations. For example, when sequencing DNA from a sample, it's generally assumed that the sequenced fraction represents a random subset of the total microbial community within that sample. Variations in species composition and abundance might emerge depending on the specific subsample analyzed, with rarer species more likely to be unevenly identified across different subsamples [19, 20]. Furthermore, agri-food sample matrices often exhibit complexity, as they encompass unpredictable and unknown microbiota in combination with substantial quantities of eukaryotic DNA. The coexistence of diverse eukaryotic cells, novel bacterial and viral species, and pathogenic bacteria complicates taxonomic classification of metagenomic sequence data, particularly for unknown species [21]. Current databases, while extensive, are not exhaustive, with pathogenic species being disproportionately represented [22, 23]. The presence of shared genomic elements across various species adds another layer of complexity to the precise identification of specific bacterial species. Finally, targeted species may be present at relative proportions below the limit of detection of metagenomic approaches [21]. Thus, it remains unclear whether metagenomics is sufficiently robust and sensitive for use in microbial surveillance in food production.

Previous studies have applied metagenomics to evaluate AMR in various sample matrices [9, 24–33]. A recognized challenge of this approach is the difficulty linking the ARG to its respective host bacterial species, especially given that these genes often reside on mobile genetic elements transferrable between species [34, 35]. Moreover, the presence of an ARG cannot necessarily be correlated to expression of a resistance phenotype. Fitzpatrick and Walsh [25] observed a difference in the distribution of ARGs where a high abundance was observed in human microbiomes but abundance in marine and soil metagenomes varied in comparison. They concluded that there are limits to detection and identification of ARGs in complex microbiome populations, noting that ARGs may not have been detected because they were present below these limits, and that failure to detect ARGs in a metagenome does not equate to absence of ARGs. Ni et al. [36] estimated the amount of metagenomic sequencing required to fulfill the objectives of a given study. They note that prokaryotes encounter different selective pressures in different environments which may affect required sequencing depth [36]. Previous studies have suggested that 10–20 X coverage of a bacterial genome is required to reliably detect ARGs in a metagenome, particularly when using stringent cutoffs for allele detection [37, 38]. However, considering shotgun metagenomic sequencing only captures a fraction of the total community within DNA sample, it is unlikely that all organisms within a sample will be equally abundant at genome coverage above 1X.

The objectives of the current study were to determine the limit of detection (LOD) for ARGs in metagenomic samples and to compare different bioinformatic tools to evaluate proficiency in accurately assigning taxonomy or identifying ARGs in complex sample matrices, such as those found in agri-food testing. Given the inherent diversity and complexity of natural microbiomes, which frequently included uncharacterized species or strains, synthetic metagenomes with known values for species composition and ARG content were generated. This approach facilitated assessment of method performance.

## Materials and methods

To facilitate reproducibility, the commands used to run bioinformatics steps are provided in Supplementary Information File 1.

### Sequences used in synthetic metagenome synthesis

Sequences for *Enterococcus faecalis*, *Escherichia coli*, *Listeria monocytogenes*, *Klebsiella pneumonia*e, and *Salmonella enterica* serovar Heidelberg from the Ottawa Laboratory Carling Canadian Food Inspection Agency (OLC-CFIA) strain collection were selected for synthetic-metagenome creation. Where possible, different genera encoding differing target ARGs of interest were selected. Sequence data was generated for this study or obtained from public repositories as indicated in Table 1. Sequencing and assembly methods for bacterial sequences utilized to create mock-metagenomes are as described previously [37]. The metagenomic sequences used as the base for spiked-metagenome formulation were short-read Illumina HiSeq raw-read sequences (Table 1).

**Table 1:** Sequences used for synthetic metagenome creation

**Sequence Identifier (SRA)**^**a**^	Strain	Description	ARGs^b^	Resistance of Interest	ARG Target^c^	Reference
**51299**^**a**^	ATCC 51299	*Enterococcus faecalis*	*catA8, aph(3’)-IIIa, ant(6)-Ia, vanW-B, vanY-B, vanS-B, vanR-B, vanH-B, vanX-B, vanB, Isa(A), erm(B), dfrE, sat4*	Vancomycin	*vanB*	[39, 40]
**SRR25084145**	DT10023001	*Escherichia coli*	*tetB, tetA, sul1, sul2, sul3, qacEdelta1, mcr-1.1, blaTEM-1, aph(6)-Id, aph(3’)-Ia, aph(3’’)-Ib, addA2*, *bla*_EC-19_, *catA1, cmlA1, dfrA1, aadA1* (multiple copies)	Colistin	*mcr-1.1*	This study
**SRR25084104**	OLC1107	*Klebsiella pneumoniae*	*bla*_CTX-M-15_, *oqxA10*, *bla*_SHV-148_, *fosA* (multiple copies), *oqxB*	ESBL	*bla*_CTX-M-15_	This study
**SRR10830862**	CFIAFB20160069	*Listeria monocytogenes*	*tetM, fosX, bcrABC*	Tetracycline	*tet(M)*	[7]
**SRR10859129**	CFIAFB20130200	*Salmonella enterica* ser. Heidelberg	*bla*_CMY-2_	ESBL	*bla*_CMY-2_	[37]
**SRR3053167**		Beef fecal metagenome	*aad9,aadA9,aadE,ant(6)-Ia,ant(6)-Ib,ant(9)-Ia,aph(3')-IIIa,blaACI-1,blaEC-18,cblA,cfr(C),cfxA_gen,cfxA6,cmx,erm(33),erm(A),erm(B),erm(C),erm(G),erm(Q),erm(T),erm(X),lnu(AN2),lnu(C),lnu(G),mef(A),mef(En2),mph(B),msr(D),sat4,spw,str,tcrB,tet(32),tet(33),tet(40),tet(44),tet(B),tet(C),tet(M),tet(O),tet(Q),tet(T),tet(W)*		NA	[26]
**SRR7414924**		Lettuce metagenome	*aac(2')-Ib,aac(2')-Ic,aac(3)-IV,aac(6')-Ie_fam,aacA-STR-10,aacA34,aadA11,aadA2,aadA6,ant(3'')-IIc,ant(6)-Ia,aph(3')-IIa,aph(4)-Ia,aph(6)-Id,aph(6)-Smalt,BcII,bla1,blaADC-151,blaCME-1,blaIND-9,blaL1,blaOXA-308,blaOXA-571,blaOXA-60,blaOXA-658,blaSPU-1,blaTEM-123,bleO,catA9,cfr-Cb,cipA,cmlR,cmx,erm(A),erm(X),erm(X),estDL136,floR,fosB_gen,fosB-251804940,lsa(B),mecI_of_mecC,mef(A),mgt,mphL,msr(D),oleD,oqxB12,oqxB16,otr(A),rgt1438,rox,rph,rphC,rphD,sul1,sul2,tet(C),tet(G),tet(O),tet(V),tetA(P),vanA-Pa,vanI,vanJ,vanK-Sc,vanM,vanO,vanR-A,vanR-O,vanS-O,vanX-Sc,vga(B)*		NA	[41]

*Abbreviations*: *SRA* sequence read archive, *ARG* antimicrobial resistance gene, *ESBL* extended spectrum-β-lactamase, *ATCC *American Type Culture Collection

^a^Data for ATCC 51299 strain (Catalog Number: 51299) is not available through the SRA. Raw sequence data locations for ATCC strains can be found on the ATCC-Bioinformatics github [40]

^b^(multiple copies) is listed next to gene(s) which were detected in multiple locations within the isolates’ genome. Isolate sequences’ AMR results are for genes with ≥ 80% template coverage. Beef and lettuce metagenome ARGs include all hits from analysis of raw-sequence data (1.0% coverage to 100% coverage)

^c^The target ARG encoded by corresponding isolate that is focused on in this study

### Synthetic metagenome construction

Synthetic metagenomes were constructed by simulating reads from assembled genomes of the five different ARG encoding organisms described in Table 1 and combining them at different coverage levels. These synthetic metagenomes were then shuffled into publicly available beef fecal and lettuce metagenomic datasets (Table 1). Synthetic metagenomes were analyzed both on their own, and after spiking into metagenomic datasets.

Illumina HiSeq short reads were synthesized from the draft genome assemblies and raw reads of the bacterial genomes using the FetaGenome2 (fabricate metagenome) tool developed in house [42]. Briefly, Art version 2.5.8 was used to simulate paired-end HiSeq reads of 150 bp in length with a 300 bp insert size. To simulate variability in coverage levels (e.g. higher coverage in plasmids vs chromosomal sequences), the FetaGenomePlasmidAware edition uses BWA to map reads to the original assembly to determine coverage depth of each contig in the given assembly, then uses the coverage report output to create more reads for higher-depth locations and fewer reads for low-depth locations of the genome. Reads were subsampled 10 times to 0.1-, 1-, 2-, 5-, and 10-X genome coverage for the bacterial genomes (Table S1). Fifty total samples (*n* = 50, Table S2) were prepared by creating ten replicates of five distinct mixtures. Each mixture consisted of varying coverage levels of the five bacteria listed in Table 1. All replicates of all synthetic mixtures were then mixed into the lettuce and beef metagenomes (Table 1). This spiking was conducted by first concatenating the replicate synthetic mixtures with the beef and lettuce metagenomes; followed by shuffling the reads using fastq-shuffle [43] with the randomseed (-r) setting activated [43]. Overall, this created 100 synthetic spiked-metagenome replicates (50 of each beef and lettuce) and 50 control synthetic-bacterial communities for analysis.

### Taxonomic profiling

Taxonomy of all synthetic metagenomes was inferred using Kraken2 version 2.1.1 [44, 45] and both Metaphlan versions 3 and 4 [46, 47]. Kraken2 analysis was conducted with the prebuilt standard PlusPF (plus plant and fungal) database [48]. After running Kraken2, Bracken (Bayesian Reestimation of Abundance with KrakEN) [49] was run at the species level to re-estimate the taxa abundance in the synthetic metagenomes using the taxonomic assignment reports from Kraken2. Reports from Kraken2/Bracken were converted to BIOM-format using kraken-biom [50] for use with Phyloseq [51] in R statistical software version 4.0.2 (R Core Team, 2014). Metaphlan3 and Metaphlan4 analyses were run using the CHOCOPhlAn 3 version v30_201901 and CHOCOPhlAnSGB vOct22_202212 marker gene databases, respectively, with default parameters to include absolute abundances.

### Statistical analysis of taxonomic classifiers

All statistical analyses were conducted using R statistical software version 3.6.3 [52]. For taxonomic assignment analysis, the increase in the number of operational taxonomic units (OTUs) assigned to target genera as a function of coverage was determined. From the Kraken2 output, the number of OTUs assigned to each of the top 5 non-target genera (*Bacillus*, *Citrobacter*, *Enterobacter*, *Shigella*, *Staphylococcus*) was also calculated and plotted with each target genus. As there were some zero-values present, a pseudocount of 0.1 was added to the number of OTUs for all data to allow log transformation. For each target-genus, a linear regression model with logarithmic transformation of both $y$ and $x$ (formula = ${\text{log}}10(y+0.1) \sim {\text{log}}10(x) * Genus$) was fit to determine the relationship between sequence coverage (covariate) and the number of assigned OTUs (outcome variable) for each of the target/non-target genus combinations. From the models, pairwise comparisons of the slope of the regression model for OTUs versus coverage were conducted using the lstrends command followed by the pairs functions from the Least-squares Means R package (formerly lsmeans, now emmeans) [53, 54].

Comparison between expected taxonomy and the classifiers Bracken, Kraken2, and Metaphlan3/Metaphlan4 was also conducted using R version 3.6.3 [52]. L2 distances (Euclidean distance) of abundances were calculated between each taxonomic classifier and expected abundance values for each genus or mix. Principal coordinate analyses were conducted including all replicates (*n* = 10) using the packages plyr [55] and phyloseq [51] with Bray–Curtis dissimilarity index and principle coordinate analysis (PCoA) ordination method.

### Antimicrobial resistance gene detection

For each synthetic-metagenome replicate, raw-reads were analysed for ARGs using the *k*-mer alignment (KMA) tool version 1.42 [56], short read sequence typer version 2 (SRST2) [57], and CARD-RGI (Comprehensive Antibiotic Resistance Database – Resistance Gene Identifier) version 5.2.1 using the protein homolog model [58, 59]. Both KMA and SRST2 were run using the NCBI AMRFinderPlus Reference Gene Catalog AMR CDS database version 3.10 (downloaded from the NCBI FTP server on 2019–11-01).

#### KMA

KMA version 1.42 with default settings was used for database indexing (NCBI AMRFinderPlus database described above) and detection of ARGs in paired-end raw reads. KMA analysis was also conducted on all subsampled isolate sequence replicates, prior to mixing, using the extended features (-ef) flag to output the mapped read counts for each ARG template.

#### SRST2

Database clustering for use with SRST2 version 0.2.0 was conducted according to authors’ instructions [60] using Cd-hit [61]. For ARG detection with SRST2 minimum coverage was set to 1, and all other settings were left at default.

#### CARD-RGI

CARD-RGI version 6.0.0 was installed via conda. The CARD database version 3.2.2 was downloaded and annotated for use with RGI according to authors’ instructions [59, 62]. RGI analysis of synthetic metagenomes was conducted using the unpublished (currently under beta-testing) RGI bwt algorithm with KMA aligner and the CARD reference sequence database.

### AMR data analysis

From KMA analysis of subsampled sequences, the read count data included in the mapstat files were merged using a custom python script based on the merge script from Metaphlan [63]. To perform ordination, data was imported into R version 3.6.3 as a phyloseq object using a custom function [63] based on the metaphlanToPhyloseq function by Wipperman [64]. Ordination of read counts mapping to ARGs for the subsampled *Enterococcus*, *E. coli*, and *Klebsiella* replicates was conducted using non-metric multi-dimensional scaling (NMDS) and Bray–Curtis dissimilarity. Reported ARG outputs from KMA, SRST2, and CARD-RGI analysis of synthetic metagenomes were enumerated, and categorized as target-gene, target-allele (eg. an allele closely related to the target gene), and non-target.

### Data availability

Raw paired-end sequence data for synthesized metagenomes have been deposited to the SRA under BioProject PRJNA922558 (Table S2). Paired-end raw reads for bacterial isolates used to synthesize mock-metagenomes are also available (Accessions in Table 1).

### Estimation of number of reads required for ARG detection

To estimate the ratio of target-isolate reads to metagenome reads needed for detecting ARGs at 5- and 10-X isolate coverage, we used a straightforward model assuming the "best-case scenario." This model assumes that all reads within a metagenome are derived from bacteria. Estimates for 5- and 10-X coverage of 3, 4, and 5 Mbp (million base pairs) isolate genomes were calculated (see equations below) and the required ratio (abundance %) of each for detection in metagenomes of 5, 10, 40, 50, 100, and 125 Mbp, with read length of 150 bp, were determined (Table 2).

**Table 2:** Bacterial isolate-derived sequencing read abundance (%) in metagenomes of varying sizes for detection of antimicrobial resistance genes in isolates with 3, 4, or 5 Mbp genomes

Metagenome Size^a^ (M)	Isolate Percentage in Metagenome^b^					
	**3 Mbp**		**4 Mbp**		**5 Mbp**	
	**5X (100000)**	**10X (200000)**	**5X (133333)**	**10X (266666)**	**5X (166666)**	**10X (333333)**
**5**	2.00	4.00	2.67	5.33	3.33	6.67
**10**	1.00	2.00	1.33	2.67	1.67	3.33
**40**	0.25	0.50	0.33	0.67	0.42	0.83
**50**	0.25	0.40	0.27	0.53	0.33	0.67
**100**	0.1	0.20	0.13	0.27	0.17	0.33
**125**	0.8	0.16	0.11	0.21	0.13	0.27

*Abbreviations*: *M* million, *Mbp* million base pairs

^a^Metagenome size refers to number of reads in metagenome

^b^For each genome size (3, 4, and 5 Mbp) 5- and 10-X genome coverage is estimated for read length of 150 bp (with number of reads to create specified coverage level in parentheses). Percentages are corresponding to metagenome size in the first column

$$Read\;count=\frac{genome\;coverage\;\times\;genome\;size}{read\;length}$$

$$Required\;isolate\;abundance=\left(\frac{Read\;count}{Total\;\#\;of\;reads\;in\;metagenome}\right)\times100$$

## Results

### Incorrect taxonomic assignment of genera in subsampled isolate whole-genome sequences due to close relatives

**Fig. 1:**
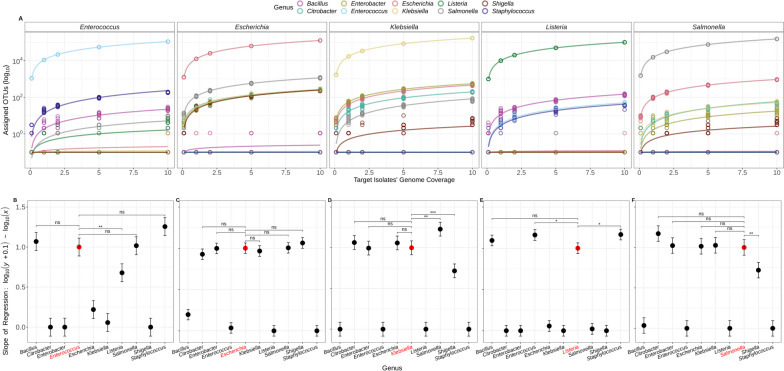
Incorrect assignment of operational taxonomic units (OTUs) to closely related genera. **A** Assigned OTUs (y-axis) as a function of target isolate’s genome coverage (x-axis). Analyses were conducted on subsampled reads of each target-genus (top-panel headings) and grouped by genus (color legend). For each coverage level (0.1, 1, 2, 5, or 10X) *n* = 10 subsampled replicates of the target organism were created. Lines represent the linear regression (log (y + 0.1) ~ log(x)) fit to each genus (see legend). **B** to **F**: Pairwise comparisons between top 10 genera with mapped OTUs and subsampled targets: B. *Enterococcus*, **C**. *Escherichia*, **D**. *Klebsiella*, **E**. *Listeria*, and **F**. *Salmonella*. Points represent the modelled slope of the regression analysis ± 95% confidence intervals (y-axis). Target organism is indicated by a red circle and red text (x-axis). Significance values are displayed above select data points of interest: *p* > 0.05 = ns; *p* < 0.05 = *; *p* < 0.01 = **; *p* < 0.001 = ***

Taxonomic assignment tools (Kraken2/Bracken, Metaphlan3/Metaphlan4) were initially assessed using synthetic sequencing read sets generated from subsampling single isolate whole-genome sequences. Taxonomic assignment conducted using Kraken2 with the standard plusPF database resulted in incorrect detection of multiple genera in single isolate sequences (Fig. 1, Figure S1). The top 10 non-target bacterial genera reported by Kraken2 included *Bacillus*, *Citrobacter*, *Enterobacter*, *Shigella*, and *Staphylococcus* (Fig. 1A, Figure S1).

To determine whether incorrect read assignment occurred due to tools detecting and reporting gene markers of closely related genera, the number of reads reported for the target organism (i.e. the subsampled isolate) were compared to the number of reads reported for each of the top non-target organisms (*Bacillus*, *Citrobacter*, *Enterobacter*, *Shigella*, and *Staphylococcus*). This was conducted by plotting the number of reported OTUs for each genus as a function of each subsampled target isolate’s genome coverage (Fig. 1A). A linear model was applied to the relationship between coverage level and OTUs for each genus, and the slopes of these relationships for each target genus:non-target genus combination were compared. This investigation sought to determine if the number of assigned OTUs for non-target organisms rose in tandem with increased coverage of the target organism. Essentially, if the slopes of the model's fit for both target and non-target aren't significantly different, it suggests that as the target's coverage expands, there's a concurrent increase in OTUs misassigned to similar non-target organisms.

The best-fitting model for the relationship between coverage level and OTUs for the target genus was a log–log linear regression (log(y) ~ log(x)) with equation $y= {b}_{0 }+{b}_{1}{x}_{1}$, and R^2^ value of ≥ 0.99 for all target genera. This model was fit for all genera including the non-targets in the subsampled isolate sequence data (Fig. 1A). For each target organism, the difference between the slope of the regression for the target and at least one non-target organism was not significant, indicating an increased detection of non-target OTU assignments as the target’s coverage expanded (Table 3, Fig. 1). For instance, subsampled reads from *E. coli* were often misidentified as related *Enterobacteriaceae* including *Citrobacter*, *Enterobacter*, *Klebsiella*, *Salmonella*, and *Shigella*. As a result, the estimated slopes for *E. coli* and these non-target species showed no significant differences (Table 3), demonstrating that as the sequencing depth of a particular target organism like *E. coli* increases, there is a concomitant rise in the number of reads incorrectly assigned to closely related genera.

**Table 3:** Comparison of linear model fit between target and non-target genera

Target genus^a^	Non-target genus^b^	Model equation^c^	*p*-value^d^
***Enterococcus**** ŷ* = 4.03 + 1.00*x*	*Bacillus*	*ŷ* = 0.276 + 1.07*x*	*p* > 0.997
	*Staphylococcus*	*ŷ* = 1.11 + 1.26*x*	*p* > 0.051
	*Salmonella*	*ŷ* = -0.304 + 1.02*x*	*p* > 0.999
***Escherichia**** ŷ* = 4.09 + 1.00*x*	*Citrobacter*	*ŷ* = 1.48 + 0.928*x*	*p* > 0.848
	*Enterobacter*	*ŷ* = 1.39 + 0.999*x*	*p* = 1.0
	*Klebsiella*	*ŷ* = 1.46 + 0.967*x*	*p* > 0.999
	*Salmonella*	*ŷ* = 1.05 + 1.01*x*	*p* = 1.0
	*Shigella*	*ŷ* = 1.34 + 1.06*x*	*p* > 0.931
***Klebsiella**** ŷ* = 4.22 + 0.999*x*	*Citrobacter*	*ŷ* = 1.22 + 1.06*x*	*p* > 0.988
	*Enterobacter*	*ŷ* = 1.74 + 0.995*x*	*p* = 1.0
	*Escherichia*	*ŷ* = 1.6 + 1.06*x*	*p* > 0.993
***Listeria**** ŷ* = 4 + 1.00*x*	*Bacillus*	*ŷ* = 1.07 + 1.09*x*	*p* > 0.595
	*Enterococcus*	*ŷ* = 0.543 + 1.16*x*	*p* < 0.024 *
	*Staphylococcus*	*ŷ* = 0.457 + 1.17*x*	*p* < 0.016 *
***Salmonella**** ŷ* = 4.18 + 1.00*x*	*Citrobacter*	*ŷ* = 0.584 + 1.17*x*	*p* > 0.282
	*Enterobacter*	*ŷ* = 0.22 + 1.02*x*	*p* > 0.999
	*Escherichia*	*ŷ* = 1.96 + 1.01*x*	*p* > 0.999
	*Klebsiella*	*ŷ* = 0.683 + 1.03*x*	*p* > 0.999

^*^Results for *Listeria* versus *Enterococcus* and *Staphylococcus* were slightly significant, but are still displayed

^a^Equation for the linear log–log model for relationship between coverage level and operational taxonomic units is below each genus

^b^For each genus in the first column, only non-target genera with interesting (non-significant) results are listed

^c^Equation for the log–log linear model fit to the relationship between coverage level and assigned operational taxonomic units for corresponding non-target genus

^d^*p*-value following statistical comparison of slopes between target genus and non-target genus. Non-significant results are displayed (*p* > 0.05); *p* < 0.05 = *

Bracken analysis of the Kraken2 reports for the subsampled isolate sequences assigned fewer OTUs to non-target organisms. *Bacillus* was not present in the top 10 genera of Bracken analyses of *Listeria* and *Enterococcus* reads and was instead replaced by the genus *Priestia*, which is also of the *Bacillaceae* family (Figure S2). Although the relationship between coverage and assigned OTUs appeared to be similar for non-target and target organisms (Figure S2), most of the models for non-target and target organisms were significantly different for Bracken outputs (Figures S2 and S3). Non-significant differences were observed for the non-targets *Listeria* and *Priestia* from subsampled *Enterococcus* reads; as well as between *Citrobacter* and *Salmonella* (Figure S2)*.* In contrast, analyses by Metaphlan3/Metaphlan4 were more specific, and did not report any non-target organisms in the subsampled sequences.

### Taxonomic assignment of genera in synthetic-metagenome mixtures

**Fig. 2:**
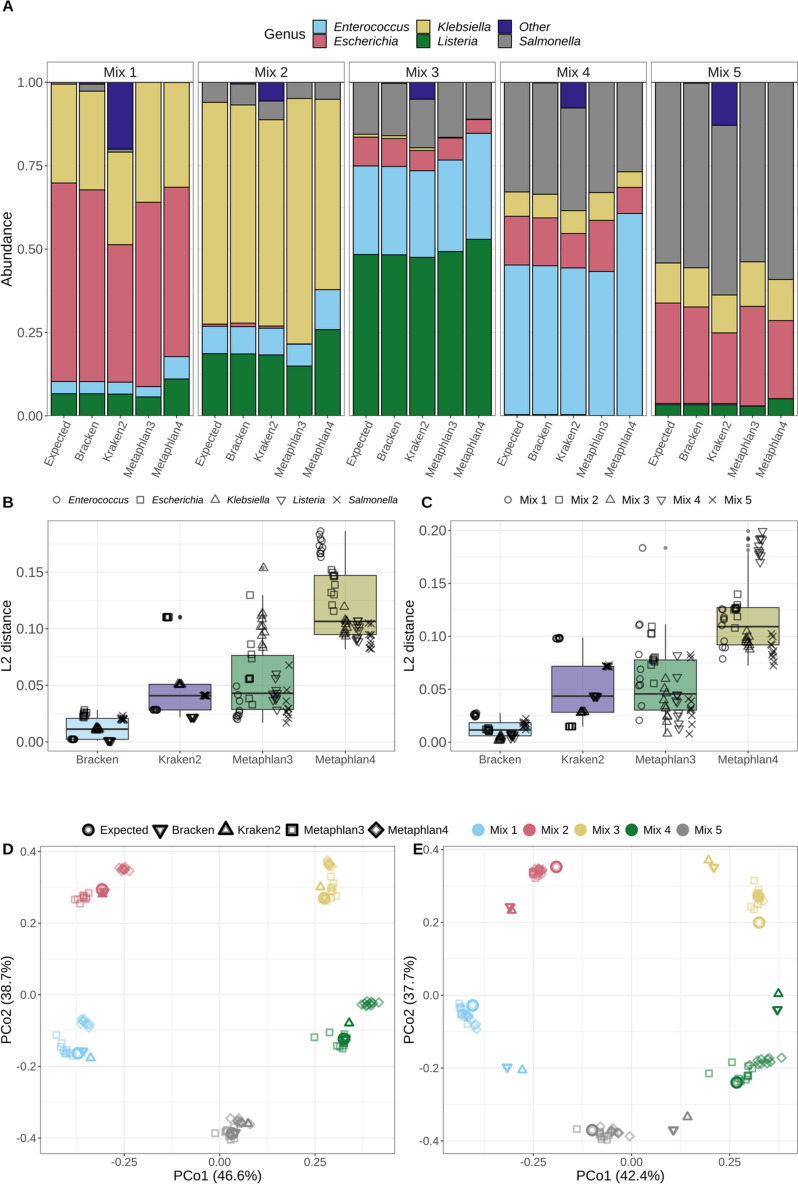
Taxonomic assignment of control mixtures by different bioinformatics tools. **A** Abundance (y-axis) of each genus (see color legend) in synthetic-community mixtures. Data for expected values are plotted next to results (average of 10 replicates) from analyses by Bracken, Kraken2, Metaphlan3, and Metaphlan4 classifiers. **B**, **C** Distance between the abundance profile for each classifier compared to the expected composition (*n* = 10 replicates). **B** L2 abundance distances for each taxonomic classifier compared to the expected composition, assessed for each genus. Genera are differentiated by point shape. **C** L2 abundance distances for each taxonomic classifier compared to the expected composition, assessed for each synthetic-community mixture. Synthetic-community mixtures are differentiated by point-shape. **D**, **E** Principal coordinate analysis of all synthetic-metagenomic mixture replicates’ (*n* = 10) (**D**) calculated organism abundances and (**E**) assigned number of operational taxonomic units. Mixtures are differentiated by colour. Point shape denotes classification method. The percentage in parentheses on each axis gives the estimated contribution of each principal coordinate to the total variance

Following analysis of the subsampled sequences from isolate genomes, synthetic metagenomes were created by mixing subsampled sequences from each of the five pathogens (*n* = 10 replicates, five combinations) (Table S2), and were then analysed for taxonomic composition and ARGs using various bioinformatic tools. Similar to the single isolate sequence analysis, Metaphlan3/Metaphlan4 analyses were the most specific, reporting only the target genera even at high organism abundance. However, Metaphlan3/4 analyses were less sensitive for organism detection. Whereas Kraken2/Bracken reported *Klebsiella* even when it was present at low levels (Mix 3 replicates), Metaphlan3 and Metaphlan4 assigned OTUs to *Klebsiella* in only two and four (respectively) of the ten low-coverage replicates even though this organism was present (Fig. 2A, Mix 3).

Abundance estimation of genera in the synthetic metagenomes by Bracken was closest to expected values as determined by L2-distance and principal coordinate analysis (PCoA) (Fig. 2 B to E). L2-distances between expected genus abundance and reported genus abundance by Bracken and Kraken2 were almost identical for all replicates (Fig. 2 B and C). In contrast, both L2-distance and PCoA for expected values versus Metaphlan3/Metaphlan4 reported values varied between replicates (Fig. 2 B to E).

### Coverage affects ARG content and detection

**Fig. 3:**
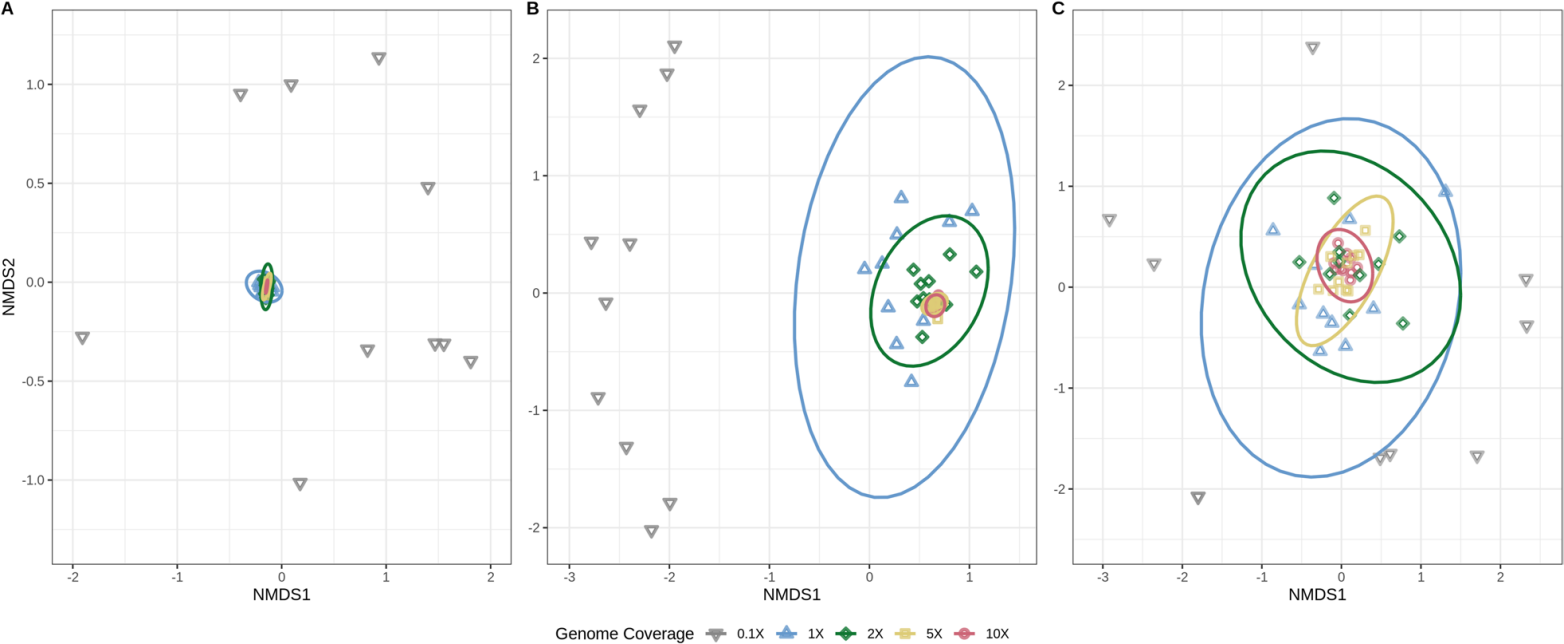
As sequence coverage increases detection of encoded AMR gene composition becomes more consistent and reliable. Non-metric multidimensional scaling (NMDS) of the number of reads mapped to AMR genes in subsampled sequence replicates for (**A**) *Enterococcus*, (**B**) *Escherichia coli*, and (**C**) *Klebsiella* isolates. Ordination was conducted using NMDS and Bray–Curtis dissimilarity. Subsampled genome coverage is differentiated by point shape and colour. *n* = 10 replicates for each of the five coverage levels (50 total per isolate). Ellipses represent 99% confidence regions. Ellipses for 0.1X genome coverage have been omitted

Analysis of the subsampled isolate sequences prior to mixing was conducted to investigate the effects of isolate genome coverage on ARG content and detection. KMA was used to determine the number of reads mapping to each ARG in the database for each subsampled replicate. Ordination was performed on the number of reads mapping to ARGs for *Enterococcus*, *E. coli*, and *Klebsiella* replicate subsamples (Fig. 3). *Salmonella* and *Listeria* were excluded as these datasets were insufficient for ordination, likely due to the low number of encoded ARGs. At lower subsampled-sequence coverage, the number of reads mapping to encoded ARGs was more varied. As sequence coverage increased, ARG composition patterns became more homogeneous (Fig. 3).

**Fig. 4:**
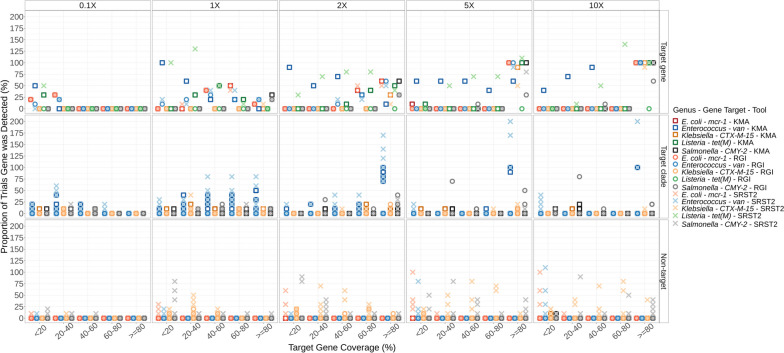
ARG detection in low complexity bacterial metagenomes. Synthetic metagenomes (*n* = 50) consisting of short-reads from five organisms mixed at different relative proportions (0.1-, 1-, 2-, 5-, and 10-X genome coverage; *n* = 10 at each coverage level) were evaluated for presence of ARGs using KMA (□), CARD-RGI (Ο), and SRST2 (✕) in silico tools. Percent ARG detection (y-axis) in 10 replicates as a function of target gene template coverage (x-axis) is shown. Point color differentiates between organism and ARG-detection tool used (see legend). Where multiple points of the same colour/shape are present for a given template-identity range (x-axis), each point represents a different allele. Detection greater than 100% indicates detection of multiple alleles, rather than only the target allele

Following analysis of individual subsampled isolate sequences, AMR analysis of the synthetic metagenome mixtures prior to spiking into the metagenomes (lettuce and beef fecal) was conducted to determine what role isolate sequence coverage played in ARG detection of a low-complexity community. Detection of ARGs of interest was divided into three categories: Target gene, refers to the target gene-allele detected in the original isolate assembly (Fig. 4, top row); Target clade, refers to detection of alleles that are within the same phylogenetic clade or closely related to the target gene (Fig. 4, middle row); Non-target refers to alleles of the target gene family that are not as closely related to the target gene (Fig. 4, bottom row). For example, *bla*_CMY-74_ (non-target) is only 90% identical to *bla*_CMY-2_ (target), whereas *bla*_CMY-44_ (target-clade) is 98.95% identical to *bla*_CMY-2_.

KMA accurately identified the target gene or closely related alleles even at low ARG target coverage (Fig. 4). Similarly CARD-RGI accurately identified most gene-alleles as the target, with the exception of *bla*_CMY-2_ which the RGI tool sometimes mapped to closely related CMY-alleles even at higher genome coverage levels (Fig. 4). In contrast, SRST2, which uses bowtie2 for read mapping, predicted non-target ARGs at ≥ 80% target coverage in some replicates, even when the isolate genome was present in the metagenome at 10X coverage (Fig. 4). For example, at 1X *Salmonella* genome coverage KMA detected *bla*_CMY-2_ at ≥ 80% target coverage in three of ten replicates and related CMY-alleles in the other seven replicates at between 40 and 79% CMY-template coverage, and one related CMY-allele at < 20% template coverage, totaling 11 predictions in the 10 replicate sequences (Table 4, Fig. 4). CARD-RGI, which utilizes KMA for target-mapping, also detected *bla*_CMY-2_ at ≥ 80% in two of ten replicates and 10 related CMY-alleles in the other eight replicates (Table 4, Fig. 4). In contrast, SRST2 detected *bla*_CMY-2_ at ≥ 80% in two of ten replicates and nine related CMY-2-alleles in the other eight replicates, but also detected several non-target CMY alleles at various coverage levels totaling 46 gene predictions in the ten replicates (Table 4, Fig. 4).

**Table 4:** Number of CMY-gene(s) and allele(s) detected by KMA and SRST2 in beef metagenomes containing *S*. ser. Heidelberg isolate present at 1X genome coverage (*n* = 10)

CMY Template Coverage	Detected ARG Relatedness to CMY-2^a^											
	**CMY-2**			**CMY-2 clade**			**Other CMY Alleles (Non-target)**			**Totals**^**b**^		
	**KMA**	**SRST2**	**RGI**	**KMA**	**SRST2**	**RGI**	**KMA**	**SRST2**	**RGI**	**KMA**	**SRST2**	**RGI**
**< 20**	-	-	-	**1**: CMY-53	-	-	-	**20**: 4 × CMY-70, 1 × CMY-83, 1 × CMY-100, 6 × CMY-157, 8 × CMY-159	-	1	20	-
**20 – 40**	-	-	-	-	**1**: CMY-44	**1**: CMY-59	-	**5**: CMY-65, CMY-74, CMY-82, CMY-100, CMY-157	-	-	6	1
**40 – 60**	-	-	-	**2**: CMY-33, CMY-130	**1**: CMY-21	**5**: 1 × CMY-59, 2 × CMY-60, 1 × CMY-59, 1 × CMY-130	-	**4**: CMY-50, CMY-65, CMY-82, CMY-90	-	2	5	5
**60 – 80**	-	-	-	**3**: CMY-44, CMY-121, CMY-132	**4**: 2 × CMY-44, 2 × CMY-161	**2:** CMY-57, CMY-59	-	**5**: CMY-68, CMY-72, CMY-89, CMY-90, CMY-114	-	3	9	2
** ≥ 80**	**3**	**2**	**2**	**2**: CMY-130, CMY-132	**3**: CMY-153, CMY-161,	**2**: CMY-61, CMY-130	-	**1**: CMY-68	-	5	6	4
**Total:**										**11**	**46**	**12**

*Abbreviations*: *KMA k*-mer alignment method, *SRST2* short read sequence typer version 2, *RGI* resistance gene identifier (by Comprehensive Antibiotic Resistance Database)

^a^Alleles detected by bioinformatic tools KMA version 1.42, SRST2, and CARD-RGI version 5.2.1 with KMA v 1.42 as the alignment method. Number in bold indicates total number of alleles detected in the 10 replicates. Enzyme-allele are listed below for each CMY-category and CMY-template coverage range

^b^Totals are listed for each CMY-template coverage category (row totals), as well as the total number of gene-alleles predicted for all 10 replicates combined for each tool (bottom row, bold number)

### ARGs present at lower coverage levels may be detected using target gene-coverage cutoffs below 80%

As genome coverage increased to 10X, ARGs were reliably detected at ≥ 80% ARG target coverage (Fig. 4). At lower isolate genome-coverage levels, the target gene was sometimes detected at a lower template coverage: for example, for *E. coli* at 2X coverage the target *mcr*-1.1 gene was detected by SRST2 at ≥ 80% in approximately 30% of trials and at 60–80% target-gene coverage in approximately 20% of trials (Fig. 4, ✕). At lower isolate-genome coverage levels, alleles closely related to the target gene were sometimes detected at a lower template coverage. For example, at 0.1X coverage, KMA detected the CMY-2 clade *bla*_CMY-61_ allele (99.91% identity to CMY-2) in one replicate at 40 – 60% target template coverage but did not detect any alleles at ≥ 60% (Fig. 4).

### Isolate genome coverage affects ARG detection in complex or agri-food metagenomes

**Fig. 5:**
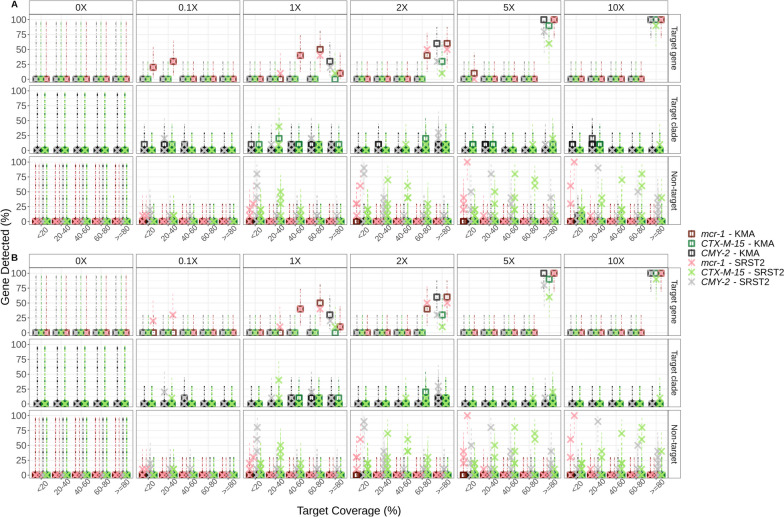
Accurate ARG detection is dependent on isolate coverage in metagenome. Synthetic metagenomes containing A) lettuce soil metagenome and B) beef fecal metagenome mixed with synthetic-community mixed reads at 0.1-, 1-, 2-, 5-, and 10-X genome coverage (*n* = 10 at each coverage level) were evaluated for presence of ARGs using both KMA (□) and SRST2 (✕) in silico tools. Only results for CTX-M-15, CMY-2, and mcr-1 are displayed (see colour legend). Lettuce, soil and beef fecal metagenomes without added synthetic-community reads were analysed as a control (0X panel, *n* = 1). Percent ARG detection (y-axis) of 10 replicates, with upper and lower 95% confidence intervals (dashed lines), are plotted as a function of detected ARG template gene coverage (x-axis). Target gene panel (right y-axis label, top row), refers to the gene-allele detected in the original isolate assembly; Target clade (middle row), refers to detection of alleles within the same phylogenetic clade as the target gene (e.g. a CMY-allele closely related to CMY-2); Non-target (bottom row), refers to alleles of the target gene family that are not as closely related to the target gene (e.g. ≤ 90% nucleotide identity to CMY-2). Darker point-color intensity is a result of multiple points (different gene-alleles) overlapping. Where multiple points of the same shape/colour are present (e.g. B: Bottom right: 10X – Non-target Alleles—≥ 80% coverage there are five CMY-2 ✕s), each point represents a different allele (e.g. blaCMY-81, blaCMY-83, blaCMY-90, blaCMY-97, and blaCMY-114, were all detected by SRST2 and are each denoted by separate ✕ points)

Analysis of microbial background effects on ARG detection was conducted by spiking the synthetic mock-communities into lettuce and beef fecal metagenomes (Table 1, Table S1). Focusing on *Salmonella* ser. Heidelberg, the target ARG, *bla*_CMY-2_ gene and additional CMY-alleles were not observed in the unspiked (control) metagenomes (Fig. 5, 0X panel). Coverage of the *Salmonella.* ser. Heidelberg isolate in the metagenome affected the proportion of trials that the *bla*_CMY-2_ target gene was accurately detected (Fig. 5). As genome coverage increased to 10X (Fig. 5, 10X panel), the target ARG (*bla*_CMY-2_) was reliably detected at ≥ 80% target coverage in all ten replicates using both KMA and SRST2. The *bla*_CMY-2_ gene was also detected at ≥ 80% ARG target coverage in all 5X replicates using KMA, but only eight out of ten replicates for SRST2 (Fig. 5). However, SRST2 also detected two closely related CMY-alleles at ≥ 80% in two of the 5X coverage replicates.

### Background microbiota influence ARG detection

Differences were observed between detected target-ARGs in the beef fecal metagenome versus the lettuce soil metagenome and synthetic bacterial metagenome (Fig. 5). For example, in Fig. 5 at 10X target isolate coverage KMA detected multiple CMY-2 related alleles at 20–40% target coverage in eight of ten spiked lettuce sample-replicates, but none of the spiked beef replicates (Fig. 5, 10X panels). Similarly, at 0.1X target *E. coli* isolate coverage KMA also detected the target mcr-1 gene at 18–40% coverage in five of ten spiked lettuce replicates, but in none of the spiked beef-fecal replicates (Fig. 5). Similar results were also noted for KMA at other target isolate coverage levels, however KMA never reported non-target alleles (Fig. 5). In contrast SRST2 did not exhibit noticeable differences depending on metagenome background, instead predicting the same number of target and non-target genes in all synthetic metagenome and spiked metagenome replicates (Fig. 5).

Results from KMA analysis of the unspiked synthetic metagenomes more closely resembled the results from lettuce sample analysis for detection of *mcr-1* (at 0.1X coverage), and both CMY-2 and CTX-M-15 related alleles at all coverage levels (Figs. 4 and 5). Kraken2 analysis of the unspiked beef and lettuce metagenomes found 17.74% and 18.33% of reads mapped to bacteria (respectively). Bracken estimation of abundance reported 89,007 (2.46%) of reads in the unspiked beef metagenome mapped to the order *Enterobacterales*, whereas only 30,433 (0.86%) of reads in the unspiked lettuce metagenome mapped to this order. The beef metagenome also had a higher number of reads mapping to *Aeromonadales* (0.12%) compared to the lettuce metagenome (0.07%).

### Proportion of isolate reads in a metagenome required for ARG detection

**Fig. 6:**
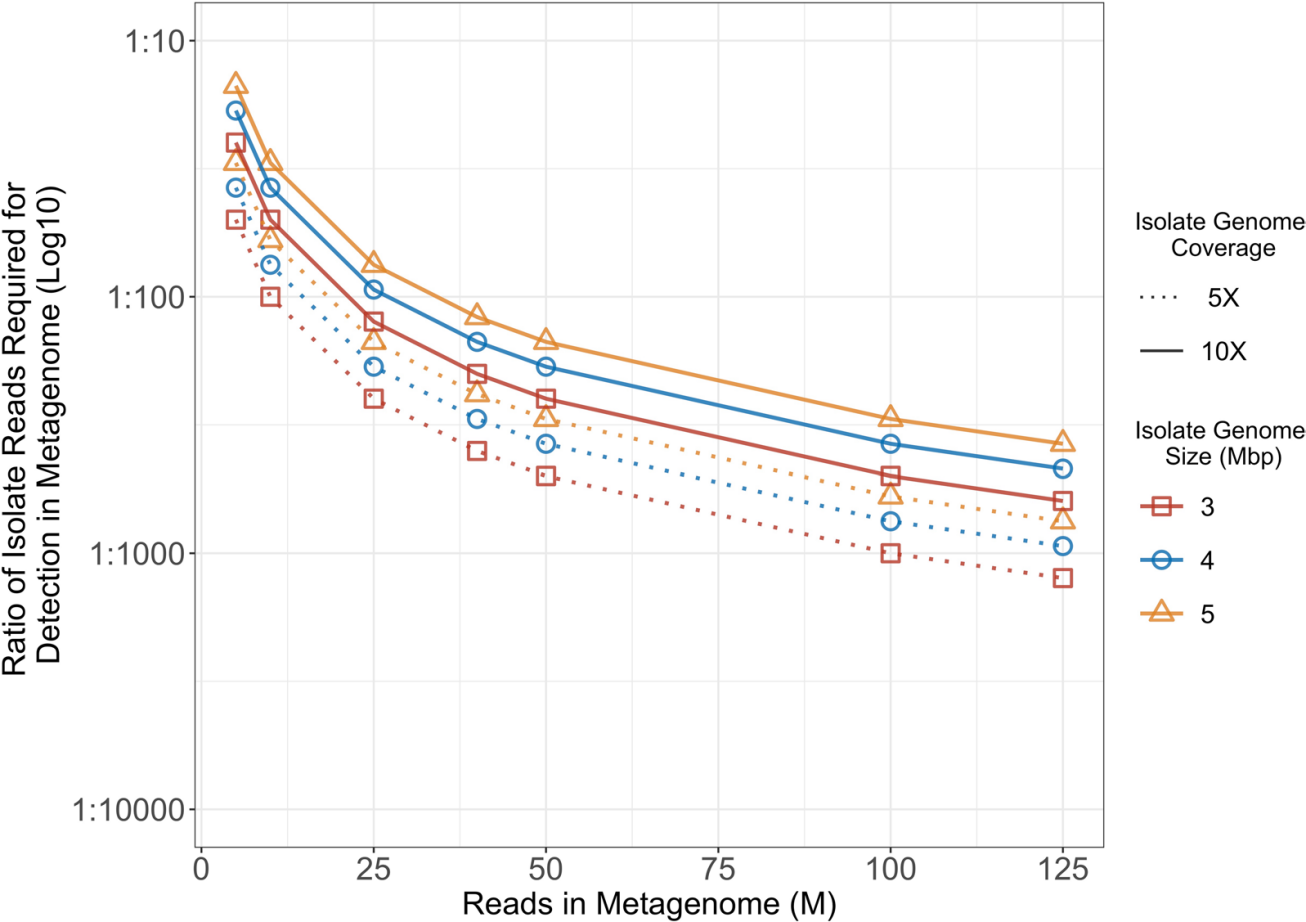
The fewer the number of bacterial reads in a metagenome, the higher the proportion the target bacteria must constitute in order to accurately detect ARGs. The ratio of isolate reads required for ARG detection in a metagenome (log10 y-axis), grouped by isolates’ genome size, was plotted as a function of total reads in metagenome (x-axis, M = million). Estimates were conducted for a “best case scenario”, where all reads in the metagenome mapped to bacteria. Isolate genome sizes of 3, 4, and 5 Mbp (million base pairs) are differentiated by point shape and colour. For each genome size (colour), isolate genome coverage levels are differentiated by linetype: 5X coverage, dotted; 10X coverage, solid

To assess the impact of relative proportion of target ARG encoding organism on ARG detection, an analysis was done to determine the ratio of isolate to metagenome reads for isolate genome sizes of 3, 4, and 5 Mbp, with genome coverage at 5X and 10X. We determined that as the total number of reads in a metagenomic sequence increased, the proportion of reads representing the isolate sequence necessary for ARG detection decreased, thereby enhancing the sensitivity of ARG detection (see Fig. 6). Notably, ARG detection was influenced by both the size of the isolate genome and the level of coverage, with smaller organisms requiring fewer reads for accurate ARG detection. In practical terms, this indicates that detection of an ARG requires that reads from an ARG encoding organism represent approximately one percent of the reads in a 25 million read metagenome and 0.1% of the reads in a 250 million read metagenome for reliable detection. Note that gene copy number and presence on mobile elements may also affect detection but was not investigated in this study.

## Discussion

Antimicrobial use in agriculture is widely believed to be one of the contributing factors to rising rates of AMR [2, 3]. As agri-food production connects many different environments and anthropogenic activities, high throughput methods enabling detection and surveillance of ARGs in agri-food samples are crucial [2, 3]. Metagenomics has the potential to be a high-throughput culture-free method enabling evaluation of the AMR within a sample. However, metagenomic sequences derived from agri-food samples are often compositionally complex and provide incomplete coverage of individual bacterial genomes [19, 20, 36]. Therefore, it is highly likely that only high-abundance organisms will be present at detectable levels and that current metagenomic techniques may not be robust or sensitive enough for detection of critically important AMR in agri-food samples, especially where the organism only constitutes an exceedingly small fraction of the sample [36, 65]. This is important because under certain conditions of selective pressure (e.g., exposure to antibiotics) a minor microbial constituency could overgrow other members of the community to become the dominant species [66].

To assess the utility of shotgun metagenomics for detection of AMR bacteria, we used synthetic metagenomes to assess the LOD for ARG detection and taxonomic classification by a variety of different bioinformatics tools. Overall, our findings indicate that reliable detection of ARGs requires exceptionally high coverage, indicating that shotgun metagenomics may be inadequate for ARG detection and surveillance. This is particularly true in situations where target organisms constitute a minor component of a microbiome and may only be present at very low coverage levels, therefore if they harbour ARGs of concern it is unlikely to be detected using metagenomics. We also found that certain commonly used tools for taxonomic assignment may exhibit inaccuracies, indicating the need for further improvements to enhance their suitability for surveillance and detection purposes.

### Taxonomic assignment

Community composition analysis relies on annotated databases; however, these databases may contain errors and pathogenic species may be over-represented in public repositories with the concomitant underrepresentation of commensal organisms such as those present in food and environmental samples [67]. Furthermore, different species can possess highly similar stretches of DNA sequences (e.g., acquired through horizontal gene transfer), leading to potential misassignments even when using a “perfect” comprehensive and accurate database [68, 69]. Following taxonomic assignment with Kraken2, an increase in detection of non-target OTUs was observed as the fold-genome coverage of the target organisms increased (Fig. 1). Other studies have investigated numerous taxonomic classifiers, including Metaphlan3/Metaphlan4 and Kraken2, using much larger metagenomic datasets [70, 71]. Our results corroborate recent findings by Johnson et al*.* [71] who reported that Kraken2 consistently misclassified high-abundance taxa thereby creating what they term “phantom” taxa, which are false-positive identification of organisms resulting from misclassification of said high-abundance taxa. These “phantom” taxa followed a similar pattern of classification to our observations for high-abundance taxa. That is, as the Kraken2/Bracken reported number of reads mapping to the target taxon increased with coverage, the number of reads mapping to the phantom taxa also increased at the same rate and therefore correlated with the target organism’s increasing coverage (Table 3). This has potential implications for those intending to compare taxonomy in their data, as the current databases are not specific for all organisms and may result in mis- or over-reporting of taxa in metagenomic samples [71].

Taxonomic classification based on read mapping tools can be hindered by the presence of closely related species. For example, *Citrobacter* exhibit high genomic similarity to *Salmonella*, with some strains having average nucleotide identities of up to 94% compared to *Salmonella* [72–74]. Similarly, *Bacillus*, *Listeria*, *Staphylococcus*, and *Enterococcus*, all belonging to the order *Bacillales*, possess gene regions that show similarities between genera [75]. Interestingly, in our metagenome analysis, we observed mis-assignments of several reads from *Enterococcus* to *Salmonella*, despite *Enterococcus* being a Gram-positive organism and *Salmonella* being Gram-negative (Fig. 1B). It is possible that taxonomy database markers may map to regions of *Enterococcus* and *Salmonella* that have similar homology. Buchrieser et al*.* [75] describe homology between gene clusters responsible for vitamin B_12_ biosynthesis in *L. monocytogenes* and *Salmonella*. However, this non-significant difference between model fit was not observed for any other Gram-positive—Gram-negative pair in our study (Fig. 1).

To evaluate taxonomic classification tools, synthetic metagenomes with a known composition were generated. Although Metaphlan3/Metaphlan4 did not misclassify reads to genera absent from the communities as Kraken2/Bracken did, abundance estimates were still closest to expected values using Bracken (Fig. 2). There are differences in the reference database types used by Bracken/Kraken2 and Metaphlan. While Bracken/Kraken2 utilizes a DNA-to-DNA method that compares reads to a comprehensive database, Metaphlan is a DNA-to-marker method where the reference database only includes specific gene families [22]. The Metaphlan3/Metaphlan4 databases, CHOCOPhlAn 3 and CHOCOPhlAn SGB 3, contain defined unique clade-level marker genes present within all strains in a clade [46, 47]. It is possible the CHOCOPhlAn 3 marker database may only include a limited number of clade-specific genes for *Klebsiella*, which resulted in lack of detection in some replicates by Metaphlan3/Metaphlan4 when *Klebsiella* was only present at 0.1X coverage (Fig. 2, Mix 3) [47]. This is likely the case for this study, as both number of assigned OTUs and abundance values determined by Kraken2/Bracken were very similar between replicates; whereas Metaphlan3/Metaphlan4 results varied greatly among replicates suggesting that the clade-specific genes were unevenly distributed among subsamples (Fig. 2B to E). Furthermore for all genera the results from Metaphlan3/Metaphlan4 differed considerably between replicates, especially at lower coverage levels (Fig. 2 B), suggesting the CHOCOPhlAn 3 markers were not mapping equally to each of these low abundance replicates. As genetic content was variable between subsampled replicates, it is possible there were no markers in the database that mapped to some of the low-coverage replicates.

### ARG detection is most accurate for highly abundant organisms

In contrast to previous work using isolate WGS data [37, 38, 76], ARG detection in a more complex sample such as an agri-food derived metagenome, is less sensitive and required lowering the stringency of target detection criteria (e.g. ≥ 80% target coverage vs ≥ 90%). We found that bacterial isolates must be present in a metagenome at an abundance sufficient to provide approximately 5- to 10-X genome coverage in order for ARGs to be accurately detected (at ≥ 80% target-gene coverage). At low coverage levels, increased variation was observed in the sequence content mapping to ARGs encoded in the subsampled sequences (Fig. 3). Although our results contrast with the 15X coverage requirements recommended by Rooney et al. [38], they utilized an assembly-based approach and were also investigating optimal sequencing depths required for detection of single nucleotide polymorphism (SNP) based resistance. Our findings are congruent with other studies which have also utilized varying sequence identity cutoffs for detecting resistomes in metagenomic sequences [3, 77, 78], and have recommended cutoffs between 80%-95% depending on desired sensitivity and stringency.

A study by Wissel et al*.* [65] to assess AMR predictions in metagenomes and reported that all ARG detection tools used performed similarly at different isolate genome coverage levels. In contrast, this study found that whereas all tools accurately predicted phenotypic resistance using isolate WGS [37], with metagenomics there is a risk of reporting false-positives for closely related ARG-alleles if the bioinformatic method used permits reads to map to multiple genes in the database, as does SRST2 which utilizes bowtie2 for read mapping (Figs. 4, 5, Table 4). This may also result in over-estimation of the ARG burden in a sample where multiple genes are reported at ≥ 80% identity but only one was actually present in the sample. At lower target-organism coverage ARGs may be detected at lower ARG target-coverage cutoffs (e.g. 40 – 60%) (Figs. 4 and 5). However, although the ARGs encoded by these low abundance organisms can be detected at lower cutoffs one must also be aware of possible detection of false-positives for alternative ARG-alleles (Figs. 4 and 5).

This study did not attempt analysis with CARD-RGI using either bowtie2 or bwa for read mapping as the creators of CARD-RGI recommend using KMA as the read aligner due “its documented better performance for redundant databases”, which are affected by the allele network problems described by Lanza et al. [79] (i.e., ARGs are closely related and often have overlapping sequence content) [62]. When using CARD-RGI in conjunction with the KMA alignment, there was a reduced detection of the *E. coli-*encoded *mcr-1* at 10X coverage. In contrast, this gene was detected in all samples by both KMA alone and SRST2 (Fig. 4). This discrepancy might be attributed to additional processing steps, like trimming, which are performed before the CARD-RGI analysis, unlike the other tools examined. Note that the CARD-RGI tool was originally created for ARG detection in isolate assemblies. The “bwt” function added to enable use of the tool with metagenomic short reads is relatively new and, as of this publication, is still under development [62]. Results from the KMA analysis of the unspiked synthetic metagenomes more closely resembled the results from lettuce sample analysis for detection of *mcr-1*, *bla*_CTX-M-15_, and *bla*_CMY-2_ related alleles at all coverage levels (Figs. 4 and 5). In comparison to the lettuce metagenome the beef metagenome encoded more bacteria of the order Enterobacterales, many of which encode chromosomal *ampC* and other β-lactam resistance genes [80–85]. It is possible that other genetic content in the beef metagenome resulted in alternative *k*-mer mismatching of these gene-alleles for the lower coverage levels (Figs. 4 and 5B) [56]. Collectively, this suggests other genetic content present in the beef metagenome could have resulted in misclassification of reads by the KMA algorithm.

Technological advancements have greatly improved DNA collection and sequencing from complex samples. Ni et al*.* [36] proposed a method to estimate the amount of metagenomic sequencing required when the abundances of different prokaryotes in a sample are known. However, in many complex sample matrices prokaryotic abundances of all organisms are not easily deduced. Even if the prokaryotic composition was known, different DNA storage, extraction, and sequencing techniques would still introduce biases in the sequence community composition (reviewed by [20, 86, 87]). As metagenomic sequencing only captures a fraction of the community within a DNA sample, it is unlikely all organisms will be equally present at high coverage levels. In fact, microbial communities within complex samples are highly uneven, with 3 – 4 orders of magnitude difference in abundance of organisms between samples of the same matrix [19, 20].

Considering a metagenome of 40 million reads where all reads are bacterial (a “clean” sample), a 5 Mbp organism would need to constitute approximately 0.8% of the metagenome to be present at 10X coverage (Table 2, Fig. 6). However, in complex matrices such as those found in agri-food production, host DNA may comprise 10 to 90% of the metagenome [21, 26, 88], and microbiome profiling becomes more inaccurate as the level of host DNA in a sample increases [21]. Therefore if only 10% of 40 million reads map to bacteria, a 5Mbp bacterial genome would have to amount to approximately 8.3% of the bacterial reads in the sample for 10X coverage enabling accurate ARG detection. One must consider the likelihood that a target organism would comprise 8% of the bacteria in a complex sample without employing significant selective protocols prior to sequencing. Minor bacterial populations that may be clinically relevant would likely be missed. For example, the major species in healthy animal feces would largely be anaerobes, and aerobic bacteria of public health significance such as *Enterobacteriaceae* could constitute as little as 0.1% of the community [89–91].

A goal of sample preparation for metagenomics is the removal of host DNA and enrichment of low abundance material such as pathogenic microorganisms [92, 93]. A single eukaryotic cell could harbor 1000 × more gDNA than a single bacterial cell, greatly impacting the relative number of informative sequencing reads. Methods for removing host DNA during extraction rely on differences in genomic DNA from eukaryotic and prokaryotic cells and can help minimize the impact of host DNA on sequencing efficiency [92–96]. While bioinformatic methods to remove host reads subsequent to sequencing have been developed, this can be challenging, particularly if a sample contains a complex mixtures of eukaryotes including plants and animals, along with microbial eukaryotes [94–98].

Previous studies have utilized metagenomics to investigate the resistome in various sample matrices including urban wastewater, cattle, animal feces, and leafy greens [21, 26, 88]. Ferreira et al. [99] compared the sensitivity of quantitative PCR (qPCR) and metagenomics for detecting ARGs in animal feces, water and wastewater samples. They reported that while metagenomics provided a markedly higher coverage of ARGs, qPCR presented higher sensitivity for ARG detection in water/wastewater, yet was not more sensitive for the fecal samples. However, for their metagenome analyses they only counted ARG sequences with 100% identity to their primer pairs as positives for comparison [99]. While studies exist investigating the LOD for AMR detection in metagenomics, many of these focus on the human microbiome or water/wastewater with few investigating methods for AMR or pathogen detection in agri-food sample types (such as animal feces and produce) [99–102].

An alternative method using targeted bait-capture techniques has been employed recently in a number of studies [79, 102–107]. In this target-baiting technique biotinylated “baits” complementary to desired target sequences (e.g. ARG sequences) are utilized to selectively bind and extract target DNA fragments from total DNA extracts. Work by Lanza et al. [79] utilized a targeted sequence capture system to analyse the resistome of human and swine fecal samples which enriched target sequence detection of ARGs 279-fold to shotgun sequencing alone. Targeted enrichment or targeted genome capture (TGC) of pathogens has also been utilized to enrich specific DNA sequences [103]. Similarly, Shay et al. [106] observed a > 300-fold improvement in recovery and detection of resistance-gene targets in retail food samples. Lee et al. [103] found a number of veterinary pathogens detected using PCR were not isolated by targeted genome capture (TGC) next generation sequencing (NGS) indicating that even enrichment approaches may not be sensitive enough for detection of clinically relevant sub-populations within a sample.

Although we were able to successfully detect the *mcr-1*, *bla*_CTX-M-15_, and *bla*_CMY-2_ genes in metagenomes spiked with synthetic-communities at 5X and 10X coverage, this was using lettuce and beef metagenomes that contained an arguably low abundance of organisms with closely related resistance genes. For example many *Enterobacteriaceae* species encode chromosomal β-lactamase resistance genes such as *bla*_ACT_ and *bla*_CMY_ alleles in some *Enterobacter* and *Citrobacter* species, respectively, which may interfere with accurate detection of clinically relevant β-lactamase genes (e.g. *bla*_CTX-M-15_, or *bla*_CMY-2_) where the genes have high homology [80–85]. Differences were observed in detection of closely related ARG-alleles the spiked beef metagenome at lower coverage levels, suggesting presence of closely related ARGs within a metagenome may affect read-mapping and should be investigated further.

## Conclusion

While shotgun metagenomics is a highly valuable technology that offers new insights into community structure along the agri-food continuum, current methodologies may not be suitable for effectively monitoring low abundance AMR bacteria in complex matrices like agri-food samples. This study highlights the necessity for at least 5X coverage of an organism to ensure reliable detection of AMR genes, making it challenging to identify organisms of concern present at low abundance (e.g., < 1% of the bacterial population) using this approach. Additionally, misclassification of sequencing reads may result in the biased misidentification of bacterial species, favoring overrepresented pathogenic species in genome databases. The potential for false-positive detection of pathogens in these samples poses a risk, as it could necessitate further investigations and subsequent actions. Nonetheless, use of these data may be appropriate under certain circumstances, and it is vital that these limitations be understood if data is to be used to inform risk assessment or for surveillance purposes.

### Supplementary Information

**Additional file 1.** Commands used for bioinformatic analyses of sequence data.

**Additional file 2:** **Table S1.** Characteristics of sequences used for synthetic-metagenome synthesis.

**Additional file 3** **Table S2.** Synthetic-community compositions.

**Additional file 4.**

**Additional file 5.**

**Additional file 6. **

## Data Availability

Raw paired-end sequence data for synthesized metagenomes has been deposited to the SRA under BioProject PRJNA922558 (Table S2). Paired-end raw reads for bacterial isolates used to synthesize mock-metagenomes are also available (Accessions in Table 1). Code for the Fetagenome-plasmidaware tool used to subsample genomes is available via github (https://github.com/OLC-Bioinformatics/FetaGenome2).
