# Supplementary material for: Modeling the limits of detection for antimicrobial resistance genes in agri-food samples: a comparative analysis of bioinformatics tools
Source: BMC Microbiol. 2024 Jan 20;24:31. doi: 10.1186/s12866-023-03148-6 (PMC10799530; doi:10.1186/s12866-023-03148-6)
Supplement: Supplementary file 1 — Additional file 1. Commands used for bioinformatic analyses of sequence data. [file 12866_2023_3148_MOESM1_ESM.docx]

**Methods – commands used for data analysis**

#### Synthetic-metagenome construction

The below command was used for each isolate bacterial sequence to subsample reads and simulate them as Illumina HiSeq reads, where bp indicates the number of bases (see publication Table 2):

**

##### Taxonomic Profiling

Kraken2 analysis was conducted with the prebuilt standard PlusPF (plus plant and fungal) database from <https://benlangmead.github.io/aws-indexes/k2> (downloaded: 2022-04-04) using the following command:

After running Kraken2, Bracken (Bayesian Reestimation of Abundance with KrakEN) was run at the species level to re-estimate the taxa abundance in the synthetic-metagenomes using the taxonomic assignment reports from Kraken2:

Reports from Kraken2 and Bracken were converted to BIOM-format using kraken-biom (<https://github.com/smdabdoub/kraken-biom>). All reports for a metagenome-type (eg. beef, chicken, lettuce, or control-mix) were converted by navigating to the folder containing sample-replicates’ reports, then using the following command:

Metaphlan3 analysis was run using the ChocoPhlAn 3 marker gene database version v30_CHOCOPhlAn_201901 with default parameters to include absolute abundances using the following command:

Metaphlan4 analysis was run using the ChocoPhlAn 3 marker gene database version vOct22_CHOCOPhlAnSGB_202212 with default parameters to include absolute abundances using the following command:

##### Antimicrobial Resistance Gene Detection

NCBI AMRFinderPlus Database

The NCBI AMRFinderPlus Reference Gene Catalog AMR CDS database version 3.10 was downloaded from the NCBI FTP server on 2019-11-01. The AMR genes were separated from the biocide and metal resistance genes into a separate multi-fasta file and formatted to serve as an AMR database for use with KMA and SRST2.

*KMA –* version 1.42

Default settings were used for database indexing and detection. KMA was used to analyse paired-end raw reads for each synthetic-metagenome using the following command:


*SRST2* – version 0.2.0

SRST2 was used to analyse paired-end raw reads for each synthetic-metagenome using the following command:

*CARD-RGI* – version 6.0.0

RGI analysis of synthetic-metagenomes was conducted using the unpublished (currently under beta-testing) RGI bwt algorithm with KMA aligner and the CARD reference sequence database using the following commands:
