# Supplementary material for: Modeling the limits of detection for antimicrobial resistance genes in agri-food samples: a comparative analysis of bioinformatics tools
Source: BMC Microbiol. 2024 Jan 20;24:31. doi: 10.1186/s12866-023-03148-6 (PMC10799530; doi:10.1186/s12866-023-03148-6)
Supplement: Supplementary file 2 — Additional file 2: Table S1. Characteristics of sequences used for synthetic-metagenome synthesis. [file 12866_2023_3148_MOESM2_ESM.docx]

Table S1. Characteristics of sequences used for synthetic-metagenome synthesis

| **Sequence^a^** | **Genome Size (bp)** | **Average Genome Coverage** | **Number of Reads^b^** |
| --- | --- | --- | --- |
|  |  |  |  |
| *Enterococcus faecalis* | 3,334,245 | 0.1 | 2223 |
|  |  | 1 | 22228 |
|  |  | 2 | 44457 |
|  |  | 5 | 111142 |
|  |  | 10 | 222283 |
|  |  |  |  |
| *Escherichia coli* | 5,431,925 | 0.1 | 3621 |
|  |  | 1 | 36213 |
|  |  | 2 | 72426 |
|  |  | 5 | 181064 |
|  |  | 10 | 362128 |
|  |  |  |  |
| *Klebsiella pneumoniae* | 5,426,509 | 0.1 | 3618 |
|  |  | 1 | 36177 |
|  |  | 2 | 72353 |
|  |  | 5 | 180350 |
|  |  | 10 | 360700 |
|  |  |  |  |
| *Listeria monocytogenes* | 3,040,020 | 0.1 | 2027 |
|  |  | 1 | 20267 |
|  |  | 2 | 40534 |
|  |  | 5 | 101334 |
|  |  | 10 | 202668 |
|  |  |  |  |
| *Salmonella enterica* serovar Heidelberg | 4,887,582 | 0.1 | 3258 |
|  |  | 1 | 32584 |
|  |  | 2 | 65168 |
|  |  | 5 | 162919 |
|  |  | 10 | 325839 |
|  |  |  |  |
| Beef fecal metagenome |  | NA | 38,251,946 |
|  |  |  |  |
| Lettuce silt metagenome |  | NA | 35,723,342 |
|  |  |  |  |
| Abbreviations: NA, not applicable (reads were not subsampled).  Abbreviations: bp, base pairs; NA, not applicable.  ^a^Beef fecal, lettuce silt, and chicken cecal metagenomes were paired-end Illumina HiSeq reads; bacterial isolate sequences were paired-end Illumina MiSeq sequences, which were used to create the paired-end HiSeq reads under the “Number of Reads” column.  ^b^Number of reads subsampled to create the corresponding “Average Genome Coverage” | | | |
