# Supplementary material for: Modeling the limits of detection for antimicrobial resistance genes in agri-food samples: a comparative analysis of bioinformatics tools
Source: BMC Microbiol. 2024 Jan 20;24:31. doi: 10.1186/s12866-023-03148-6 (PMC10799530; doi:10.1186/s12866-023-03148-6)
Supplement: Supplementary file 3 — Additional file 3 Table S2. Synthetic-community compositions. [file 12866_2023_3148_MOESM3_ESM.docx]

| Table S2. Synthetic-community compositions | |
| --- | --- |
| Synthetic-community Mixture | Organism Composition^a^ |
| Mix 1  SRA BioSample: SAMN32664128 | 0.1X *Salmonella* 1X *Enterococcus* 2X *Listeria* 5X *Klebsiella* 10X *Escherichia* |
| Mix 2  SRA BioSample: SAMN32664129 | 0.1X *Escherichia* 1X *Salmonella* 2X *Enterococcus* 5X *Listeria* 10X *Klebsiella* |
| Mix 3  SRA BioSample: SAMN32664130 | 0.1X *Klebsiella* 1X *Escherichia* 2X *Salmonella* 5X *Enterococcus* 10X *Listeria* |
| Mix 4  SRA BioSample: SAMN32664131 | 0.1X *Listeria* 1X *Klebsiella* 2X *Escherichia* 5X *Salmonella* 10X *Enterococcus* |
| Mix 5  SRA BioSample: SAMN32664132 | 0.1X *Enterococcus* 1X *Listeria* 2X *Klebsiella* 5X *Escherichia* 10X *Salmonella* |

Abbreviations: SRA, Sequence Read Archive.
^a^The X refers to coverage. Eg. 0.1X is 0.1-fold coverage.
