# Supplementary figures and images for: Modeling the limits of detection for antimicrobial resistance genes in agri-food samples: a comparative analysis of bioinformatics tools
Source: BMC Microbiol. 2024 Jan 20;24:31. doi: 10.1186/s12866-023-03148-6 (PMC10799530; doi:10.1186/s12866-023-03148-6)

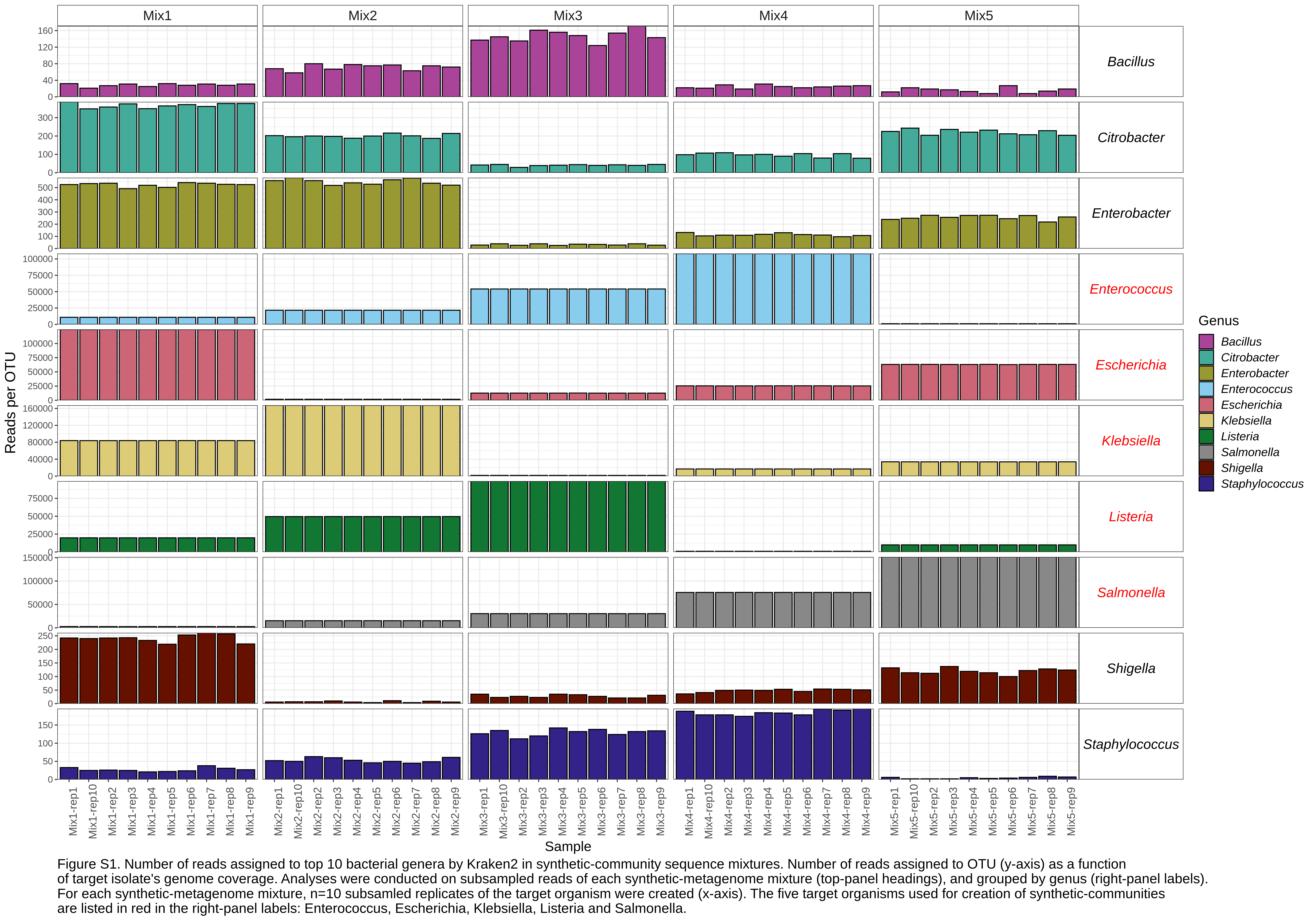

Supplement: Supplementary file 4 — Additional file 4. [file 12866_2023_3148_MOESM4_ESM.png]

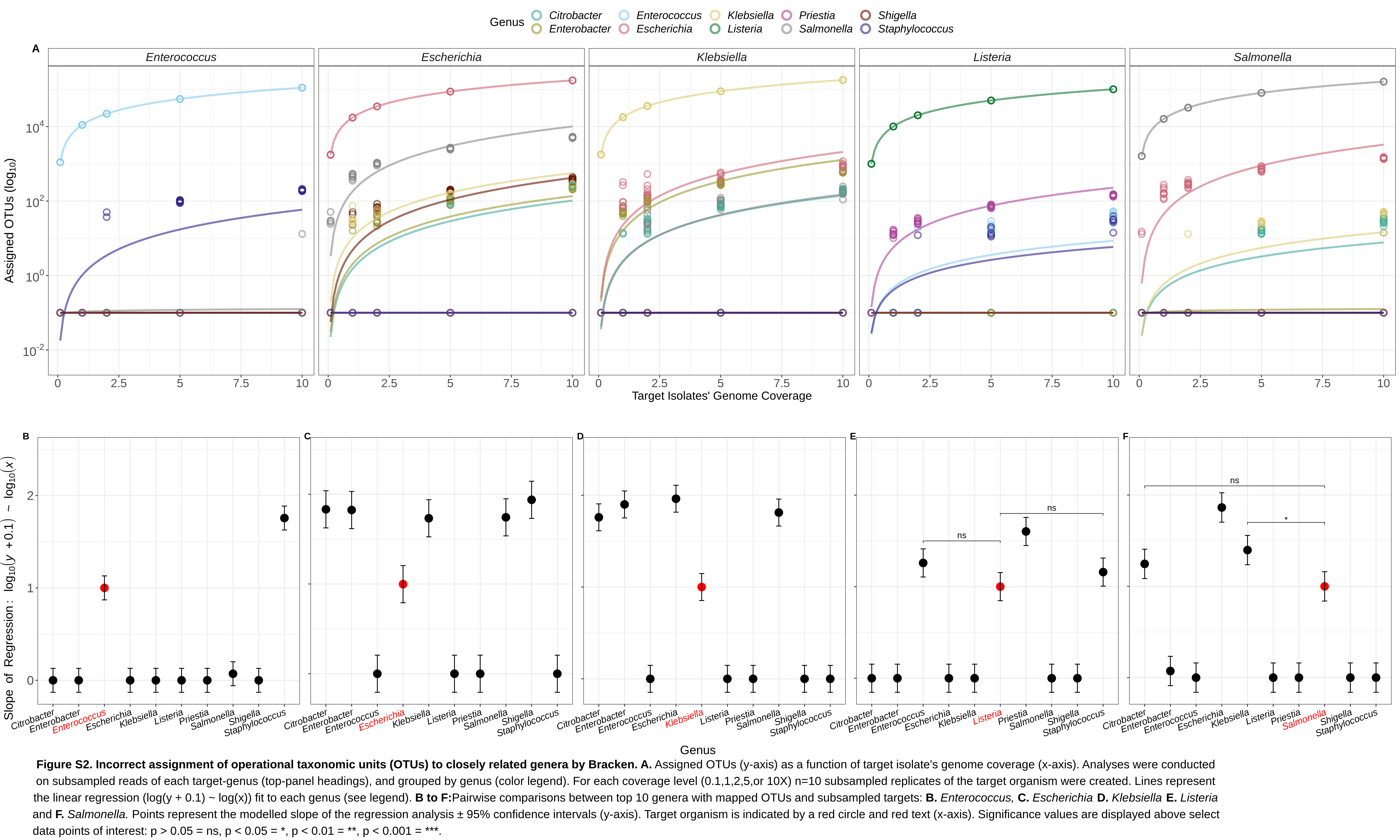

Supplement: Supplementary file 5 — Additional file 5. [file 12866_2023_3148_MOESM5_ESM.png]

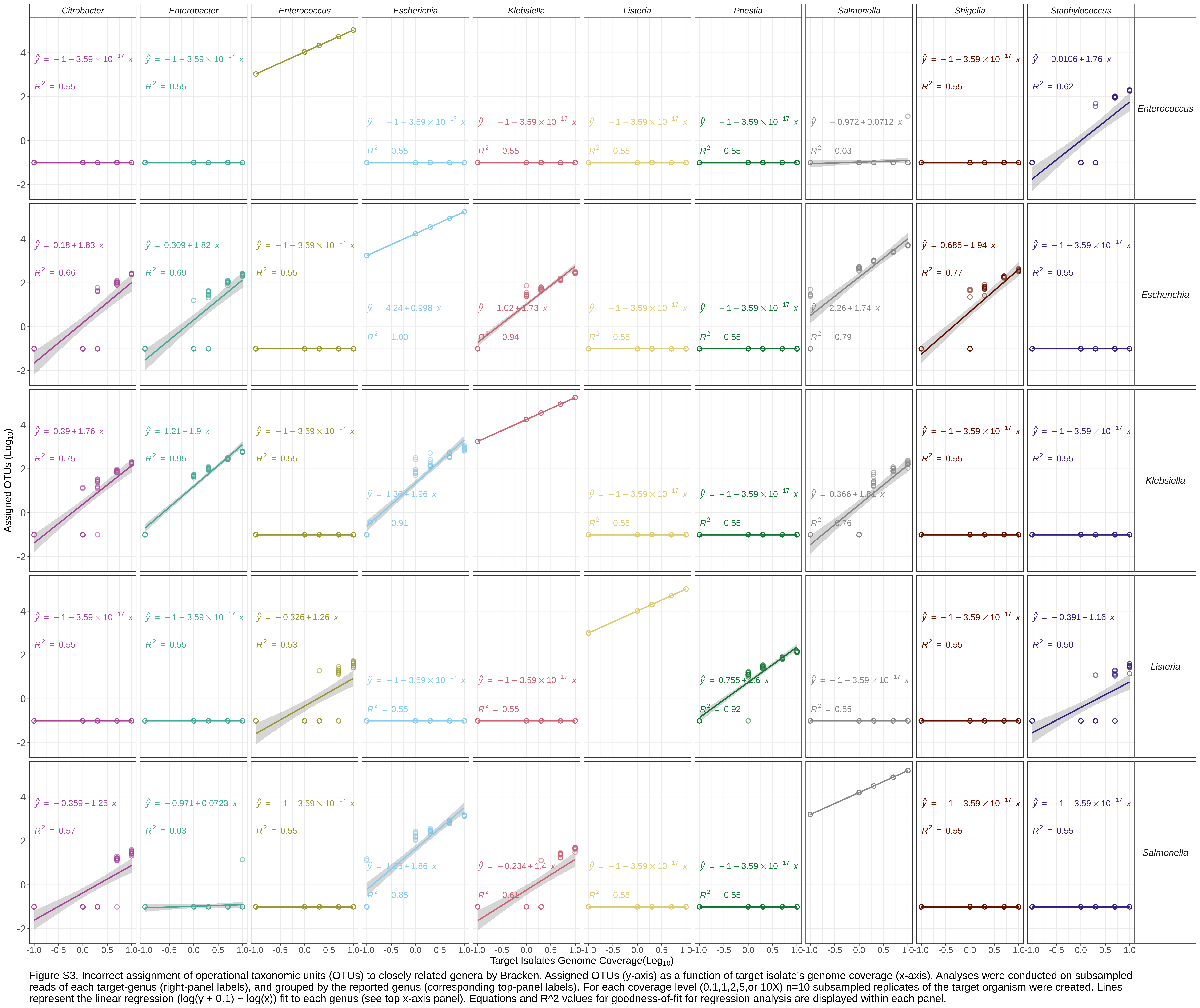

Supplement: Supplementary file 6 — Additional file 6. [file 12866_2023_3148_MOESM6_ESM.png]
